# Supplementary material for: Modeling glioblastoma heterogeneity as a dynamic network of cell states
Source: Mol Syst Biol. 2021 Sep 16;17(9):e10105. doi: 10.15252/msb.202010105 (PMC8444284; doi:10.15252/msb.202010105)
Supplement: Supplementary file 6 — Source Data for Figure 5 [file MSB-17-e10105-s004.zip › Figure5A_sourcedata/GSEA_3017/hallmarks_stateD.GseaPreranked.1621934542601/HALLMARK_DNA_REPAIR.html]

Details for gene set HALLMARK\_DNA\_REPAIR[GSEA]

|  || Dataset | state23017 |
| Phenotype | NoPhenotypeAvailable |
| Upregulated in class | na\_neg |
| GeneSet | HALLMARK\_DNA\_REPAIR |
| Enrichment Score (ES) | -0.39345613 |
| Normalized Enrichment Score (NES) | -1.5279703 |
| Nominal p-value | 0.059737157 |
| FDR q-value | 0.15432583 |
| FWER p-Value | 0.812 |
Table: GSEA Results Summary

  

Fig 1: Enrichment plot: HALLMARK\_DNA\_REPAIR      
 Profile of the Running ES Score & Positions of GeneSet Members on the Rank Ordered List

  

| PROBE | GENE SYMBOL | GENE\_TITLE | RANK IN GENE LIST | RANK METRIC SCORE | RUNNING ES | CORE ENRICHMENT || 1 | MPC2 |  |  | 178 | -0.252 | -0.1074 | No |
| 2 | POLA2 |  |  | 312 | -0.264 | -0.1787 | No |
| 3 | XPC |  |  | 362 | -0.269 | -0.1849 | No |
| 4 | POLH |  |  | 467 | -0.282 | -0.2319 | No |
| 5 | RFC5 |  |  | 568 | -0.293 | -0.2745 | No |
| 6 | RFC4 |  |  | 575 | -0.295 | -0.2445 | No |
| 7 | RFC2 |  |  | 607 | -0.302 | -0.2329 | No |
| 8 | SDCBP |  |  | 734 | -0.327 | -0.2916 | No |
| 9 | RRM2B |  |  | 839 | -0.355 | -0.3300 | No |
| 10 | RFC3 |  |  | 852 | -0.358 | -0.2973 | No |
| 11 | NME3 |  |  | 978 | -0.407 | -0.3458 | Yes |
| 12 | POLD3 |  |  | 1002 | -0.415 | -0.3149 | Yes |
| 13 | SAC3D1 |  |  | 1025 | -0.429 | -0.2815 | Yes |
| 14 | LIG1 |  |  | 1035 | -0.437 | -0.2372 | Yes |
| 15 | DDB2 |  |  | 1045 | -0.443 | -0.1922 | Yes |
| 16 | POLA1 |  |  | 1088 | -0.464 | -0.1701 | Yes |
| 17 | ZWINT |  |  | 1099 | -0.467 | -0.1231 | Yes |
| 18 | PCNA |  |  | 1142 | -0.504 | -0.0964 | Yes |
| 19 | FEN1 |  |  | 1191 | -0.569 | -0.0667 | Yes |
| 20 | DUT |  |  | 1223 | -0.633 | -0.0164 | Yes |
| 21 | TYMS |  |  | 1272 | -0.770 | 0.0369 | Yes |
Table: GSEA details [plain text format]

  

Fig 2: HALLMARK\_DNA\_REPAIR: Random ES distribution      
 Gene set null distribution of ES for **HALLMARK\_DNA\_REPAIR**
